# Equine grass sickness (a multiple systems neuropathy) is associated with alterations in the gastrointestinal mycobiome

**DOI:** 10.1186/s42523-021-00131-2

**Published:** 2021-10-09

**Authors:** Bruce C. McGorum, Zihao Chen, Laura Glendinning, Hyun S. Gweon, Luanne Hunt, Alasdair Ivens, John A. Keen, R. Scott Pirie, Joanne Taylor, Toby Wilkinson, Gerry McLachlan

**Affiliations:** 1 Royal (Dick) School of Veterinary Studies and The Roslin Institute, Easter Bush Veterinary Centre, University of Edinburgh, Roslin, Midlothian, EH25 9RG UK; 2 Ashworth Laboratories, University of Edinburgh, Edinburgh, EH9 3FL UK; 3 School of Biological Sciences, University of Reading, Reading, RG6 6EX UK; 4 Royal Botanic Garden Edinburgh, 20A Inverleith Row, Edinburgh, EH3 5LR UK

**Keywords:** Equine grass sickness, Equine dysautonomia, Gastrointestinal mycobiota, Fungi, Multiple systems neuropathy

## Abstract

**Background:**

Equine grass sickness (EGS) is a multiple systems neuropathy of grazing horses of unknown aetiology. An apparently identical disease occurs in cats, dogs, rabbits, hares, sheep, alpacas and llamas. Many of the risk factors for EGS are consistent with it being a pasture mycotoxicosis. To identify potential causal fungi, the gastrointestinal mycobiota of EGS horses were evaluated using targeted amplicon sequencing, and compared with those of two control groups. Samples were collected *post mortem* from up to 5 sites in the gastrointestinal tracts of EGS horses (EGS group; 150 samples from 54 horses) and from control horses that were not grazing EGS pastures and that had been euthanased for reasons other than neurologic and gastrointestinal diseases (CTRL group; 67 samples from 31 horses). Faecal samples were also collected from healthy control horses that were co-grazing pastures with EGS horses at disease onset (CoG group; 48 samples from 48 horses).

**Results:**

Mycobiota at all 5 gastrointestinal sites comprised large numbers of fungi exhibiting diverse taxonomy, growth morphology, trophic mode and ecological guild. FUNGuild analysis parsed most phylotypes as ingested environmental microfungi, agaricoids and yeasts, with only 1% as gastrointestinal adapted animal endosymbionts. Mycobiota richness varied throughout the gastrointestinal tract and was greater in EGS horses. There were significant inter-group and inter-site differences in mycobiota structure. A large number of phylotypes were differentially abundant among groups. Key phylotypes (*n* = 56) associated with EGS were identified that had high abundance and high prevalence in EGS samples, significantly increased abundance in EGS samples, and were important determinants of the inter-group differences in mycobiota structure. Many key phylotypes were extremophiles and/or were predicted to produce cytotoxic and/or neurotoxic extrolites.

**Conclusions:**

This is the first reported molecular characterisation of the gastrointestinal mycobiota of grazing horses. Key phylotypes associated with EGS were identified. Further work is required to determine whether neurotoxic extrolites from key phylotypes contribute to EGS aetiology or whether the association of key phylotypes and EGS is a consequence of disease or is non-causal.

**Supplementary Information:**

The online version contains supplementary material available at 10.1186/s42523-021-00131-2.

## Background

Equine grass sickness (EGS) is a predominantly fatal, multiple systems neuropathy of grazing horses that kills approximately 1–2% of horses grazing affected premises in the United Kingdom annually [1–3]. It is characterised by chromatolysis, degeneration and loss of enteric neurons, peripheral and central autonomic neurons, neurons in specific brain stem nuclei and spinal cord somatic efferent lower motor neurons [3, 4]. The predominant clinical features of EGS are attributable to paralysis of the entire gastrointestinal (GI) tract caused by severe enteric neuropathy [1, 2]. Striking similarities in the clinico-pathological features of EGS with multiple systems neuropathies of cats (feline dysautonomia), dogs (canine dysautonomia), hares (leporine dysautonomia), rabbits, alpacas, llamas and sheep (abomasal emptying defect) suggest these represent a specific disease entity with a common, but currently unknown, aetiology [5–11]. While some evidence supports an association with *Clostridium botulinum* type C/D [1, 2, 12–14], many risk factors for EGS are consistent with it being caused by a neurotoxic extrolite from a pasture-derived fungus [1, 15]. Equids, being monogastric animals, are considered to be more sensitive to dietary fungi and mycotoxins than ruminants [16]. EGS has a strong association with grazing, particularly on certain pastures [17]. Pasture-risk factors for EGS include sand/loam rather than chalk soils, high soil nitrogen, low soil Cu and Zn content, and pasture disturbance [18–20]. As for other pasture mycotoxicoses, EGS is strongly seasonal, with peak incidence occurring during spring and early summer [17–21] when many fungi are actively growing [22]. The seasonality may reflect climate-level risk factors for EGS, including cooler, drier weather and irregular ground frosts [17–19], which may favour fungal growth and extrolite elaboration [19], and/or the link between the growth of causal fungi on plants and the seasonal pattern of plant growth [23]. The first description of EGS in 1906 has been linked to the dramatic pasture improvements made in Scotland at that time, including drainage, ploughing, a change from permanent to rotational grazing and introduction of new grass seed mixtures which may have been contaminated with fungal spores [24]. Previous studies identified *Acremonium* and *Fusarium* on plants from all studied EGS fields in Scotland and Patagonia [24]. Comparison of the intestinal mycobiota of EGS and control horses using culture-based techniques identified a wide variety of fungi including mycotoxin producing species in EGS and control horses, but EGS was not associated with a particular mycotoxigenic fungus [22]. These authors acknowledged significant study limitations, in particular the inability to isolate and identify the wide variety of fungi present in the equine GI tract. Mycotoxicosis may also account for the depletion of the plasma sulphur amino acids cyst[e]ine and methionine in EGS and feline dysautonomia [25, 26], since these strong nucleophiles reduce, detoxify and facilitate the excretion of electrophilic compounds and free radicals derived from dietary toxins including fungal extrolites [27, 28]. EGS was not induced in experimental horses by intra-gastric administration of cultures of fungi obtained from EGS plants including a *Basidiomycete*, *Rhizopus*, *Mucoraceae* and a putative *Phoma* [29], nor by feeding* Acremonium* spp.-colonised Festuca grass [30] or ergots [31]*.*

The aim of this study was to investigate whether EGS is associated with ingestion of mycotoxin-producing fungi. Targeted amplicon sequencing of the internal transcribed spacer 1 (ITS1) of the fungal ribosomal RNA gene cluster was used to compare the mycobiota within the stomach, ileum, caecum, colon and faeces of EGS and control grazing horses, and within the faeces of healthy co-grazing control horses. Co-grazing control horses remain clinically healthy but have increased serum concentrations of acute phase proteins [32] consistent with sub-clinical exposure to the toxin which causes EGS. Metabarcoding analysis of the mycobiota throughout the gastro-intestinal (GI) tract of grazing horses has not been previously reported. We hypothesised that detection of putative causal fungi was more likely achieved by examination of the GI mycobiota of grazing EGS horses than by examination of mycobiota of soil and herbage samples collected from the horses’ fields. This study identified key phylotypes (*n* = 56) that had increased abundance and high prevalence in EGS samples, and which were important determinants of the inter-group differences in mycobiota structure. Further work is required to determine whether neurotoxic extrolites from these key phylotypes have a causal role in EGS, or whether their association with EGS is coincidental.

## Results

### Sample groups

There was no significant inter-group difference in sex (Chi-square test *P* = 0.44) or month of sample collection (paired t-test *P* = 0.08). EGS horses (median 5 years, inter-quartile range 4.0–7.5) were significantly younger than CoG (8 years, 5.0–10.3; Mann–Whitney *P* = 0.011) and control (16 years, 12.0–25.0; *P* < 0.0001) horses.

### Sequence data

A total of 23,590,409 high quality sequences, representing 13,204 OTUs and 2816 phylotypes of diverse taxonomy, were acquired from 265 GI samples (Additional file 1; Table S1). Samples had a minimum of 5122 and maximum of 2,152,731 sequences (median 60,768; inter-quartile range 32,600–106,289; mean 89,020). Rarefaction curves of phylotype richness for individual samples (Additional file 1; Fig. S1) indicated adequate sample size to capture the complex and diverse mycobiota structure.

### Negative and positive controls

Negative (nuclease-free water) and positive mock fungal community controls were run in parallel with samples. Mock community control 1 was a mixed microbial population which included rDNA from *Saccharomyces cerevisiae* and *Cryptococcus neoformans*. Mock community 2 comprised rDNA extracted from 10 fungi (*Alternaria infectoria*, *Coniochaeta lignicola*, *Didymella rumicicola*, *Mycosphaerella tassiana*, *Penicillium pagulum*, *Pyrenochaetopsis pratorum*, *Vishniacozyma victoriae*, *Xylaria longipes*, G_*Eutypella* and G_*Fusarium*) which colonised grasses collected from equine pastures within the geographical area from which the equine GI samples were collected. Negative controls (*n* = 16) had low amplicon counts (mean 1486, range 32–9672), except 3 samples that were contaminated with *Alternaria infectoria* and *Mycosphaerella tassiana*. All 12 taxa in the two mock communities were identified in all mock community samples. 2/10 mock community 2 and 4/7 mock community 1 samples had total amplicon counts < 5000 and were removed from the analysis. *Saccharomyces cerevisiae* and *Cryptococcus neoformans* were identified in all 3 mock community 1 samples, but the former was in low abundance. All 10 fungi were identified in all mock community 2 samples. Some taxa in mock community 2, including *Coniochaeta lignicola*, were represented by a single OTU, while others, including *Vishniacozyma victoriae*, had multiple OTUs. Of the 13,204 OTUs, 53 were likely contaminants; as this analysis was done retrospectively the contaminants were not removed from the subsequent analysis. None of the contaminant OTUs contributed to the key phylotypes.

### FUNGuild analysis

FUNGuild analysis parsed 2460 of the 2816 (87%) phylotypes into 20 growth morphologies, 26 ecological guilds and 3 tropic modes. Some fungi were parsed into multiple categories. Growth morphologies were predominantly microfungi (32.7%), null (30.2%), agaricoid (14.7%) and yeast (9.5%) (Additional file 1; Table S2). Tropic mode was predominantly saprotrophs (75.3%), with smaller proportions of pathotrophs (35.0%) and symbiotrophs (25.9%) (Additional file 1; Table S3). In terms of ecological guild, most phylotypes were assigned to undefined saprotrophs (55.1%), plant pathogens (23.2%), wood saprotrophs (16.7%), endophytes (12.8%), animal pathogens (11.8%), fungal parasites (10.3%), ectomycorrhizal (8.9%), soil saprophytes (7.4%) and dung saprotrophs (6.8%) (Additional file 1; Table S4). Only 1% of phylotypes were animal endosymbionts including *Neocallimastigaceae*.

### Dominant fungal taxa

**Fig. 1:**
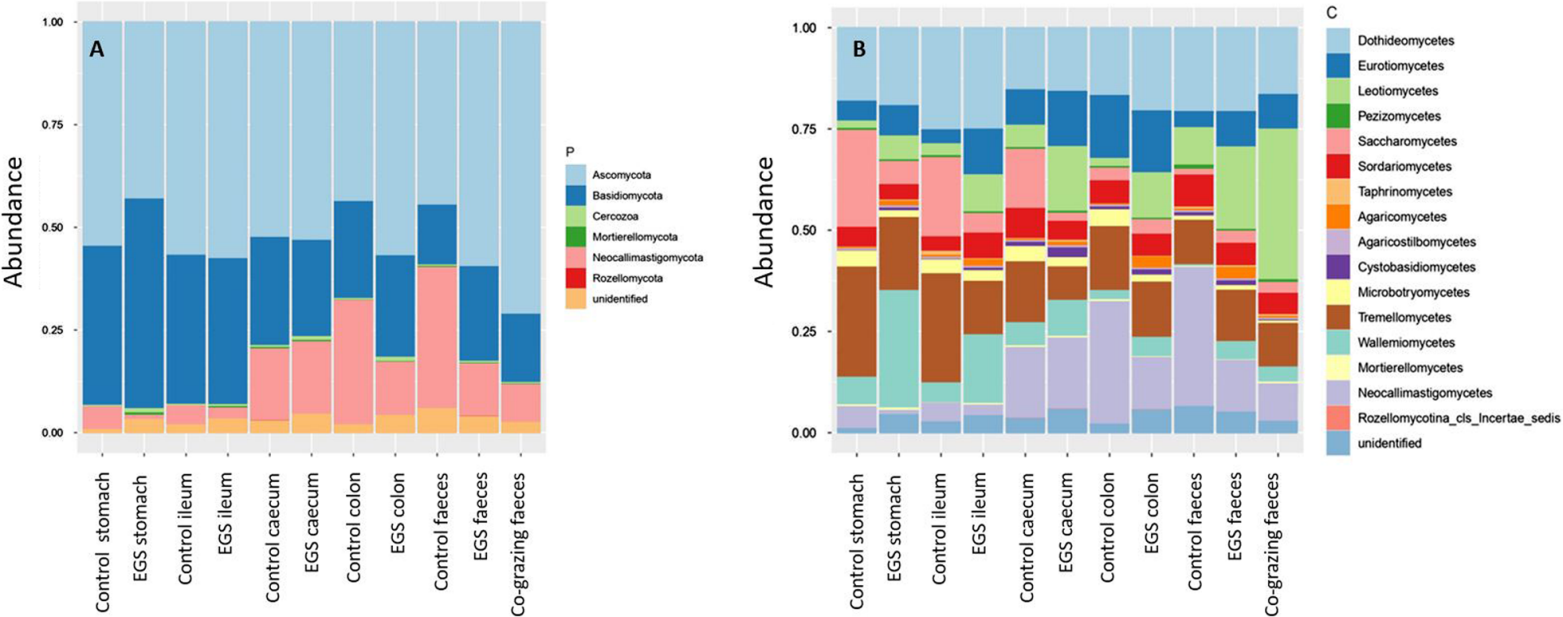
Taxonomy plots showing relative abundance of taxa at **A** phylum and **B** class levels. Data are filtered at 0.05% abundance threshold

Taxa were identified to kingdom (98.0%, *n* = 3), phylum (71.8%, *n* = 18), class (66.6%, *n* = 46), order (64.5%, *n* = 143), family (55.4%, *n* = 355), genus (50.7%, *n* = 1004) and species (40.1%, *n* = 2317) levels. Visual taxonomic summaries for all samples are presented for phylum, class (Fig. 1), order and family (Additional file 1: Fig. S2). While most taxa were k*_Fungi*, there were also protists (k_*Rhizaria*) and taxa unidentified at kingdom level. The dominant phyla were *Ascomycota*, *Basidiomycota*, *Mortierellomycota* and *Neocallimastigomycota* (Fig. 1A). Dominant classes were *Dothideomycetes*, *Eurotiomycetes*, *Leotiomycetes*, *Neocallimastigomycetes*, *Saccharomycetes*, *Sordariomycetes*, *Tremellomycetes* and *Wallemiomycetes* (Fig. 1B). Dominant orders were *Capnodiales*, *Eurotiales*, *Filobasidiales, Neocallimastigales*, *Pleosporales*, *Saccharomycetales* and *Thelebolales* (Additional file 1: Fig. S2a). Dominant families were *Aspergillaceae*, *Bulleribasidiaceae*, *Filobasidiaceae, Neocallimastigaceae*, *Phaffomycetaceae, Sporormiaceae*, *Thelebolaceae* and *Wallemiaceae* (Additional file 1: Fig. S2b). Dominant genera were *Aspergillus*, *Naganishia, Piromyces*, *Preussia*, *Thelebolus*, *Vishniacozyma*, *Wallemia* and *Wickerhamomyces*. Dominant species were *Aspergillus proliferans*, *Vishniacozyma victoriae*, *Wallemia muriae*, *W. sebi* and *Wickerhamomyces anomalus*.

### Fungal species richness and diversity (alpha-diversity)

**Fig. 2:**
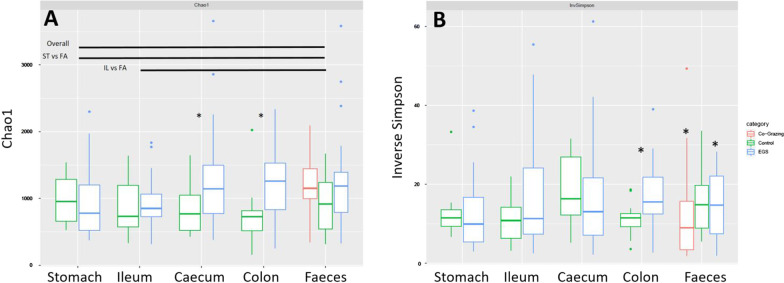
Box plots showing alpha-diversity of mycobiota in different GI sites for EGS, CTRL and CoG horses, estimated as **A** Chao1 and **B** Inverse Simpson indices. Inter-site significant differences are identified as bars, while inter-group significant differences are indicated by asterisks. For statistical analysis, see Additional file 1; Table S5). *FA* faeces, *IL* ileum, *ST* stomach

Mycobiota richness (Chao 1) differed significantly across all 5 GI sites (Kruskal Wallis; *P* = 0.0084) and among groups (Fig. 2; Additional file 1; Table S5). Mycobiota richness was higher in faeces versus proximal (stomach and ileum) GI sites. Pairwise inter-site comparisons indicated richness was higher in faeces than in stomach (Chao1 *P* = 0.00364) and ileum (Chao1 *P* = 0.00190) (Additional file 1; Table S5). Diversity (Inverse Simpson) was not significantly different across GI sites (Kruskal Wallis; *P* = 0.1914).

Inter-group comparisons demonstrated increases in Chao1 in caecum and colon samples from EGS horses versus those from CTRL horses (Table 1), and increases in Inverse Simpson index in EGS colon samples versus those from controls and in EGS faeces samples versus those from CoG horses (Table 1).

**Table 1:** Inter-group comparisons of alpha-diversity indices at each GI site

	Stomach	Ileum	Caecum	Colon	Faeces		
	EGS vs CTRL				EGS vs CTRL	EGS vs CoG	CoG vs CTRL
Chao1	0.475	0.547	**0.0179**	**0.00267**	0.165	0.699	0.1
Inverse simpson	0.590	0.295	0.360	**0.0148**	0.884	**0.0299**	0.0653

*P* values indicating significantly higher alpha-diversity in EGS samples are in bold

### Inter-site and inter-group differences in mycobiota structure (Beta-diversity)

**Fig. 3:**
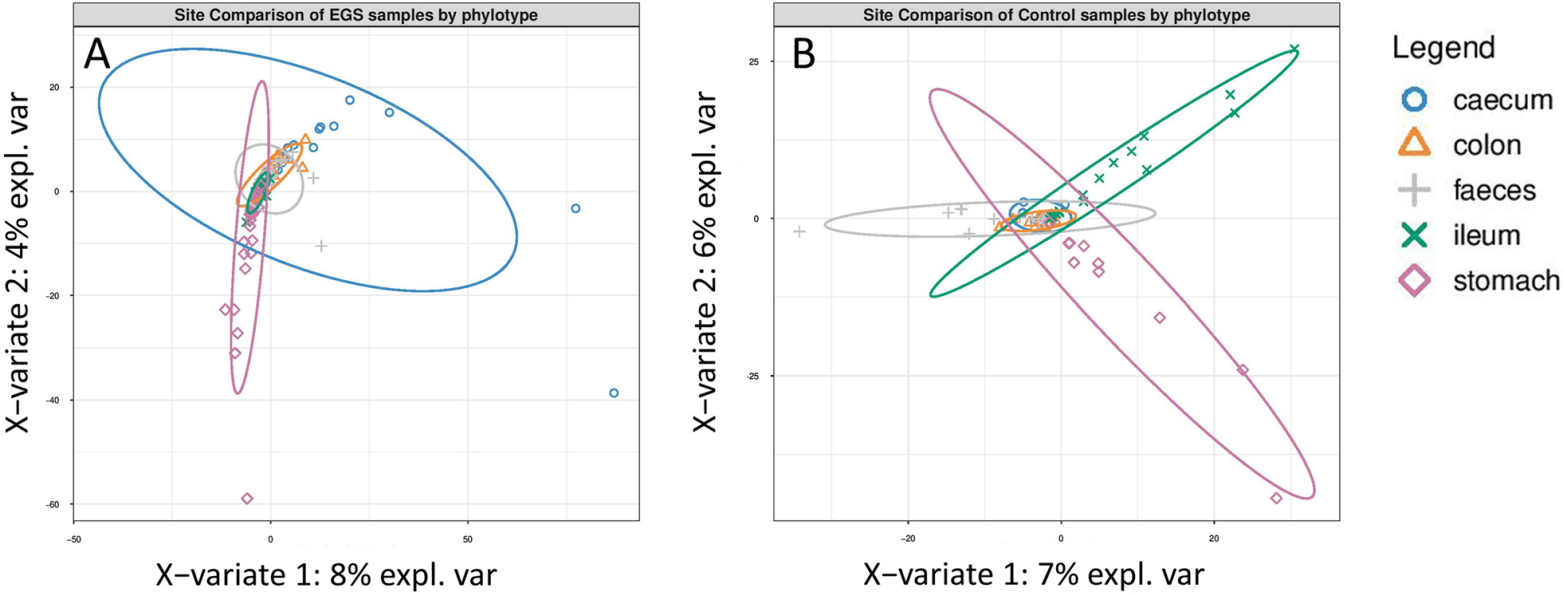
PLS-DA plots showing clustering of samples by GI site in **A** EGS and **B** CTRL groups, at phylotype level. Confidence ellipses show 95% confidence level. For statistical analysis, see Additional file 1; Table S6

**Fig. 4:**
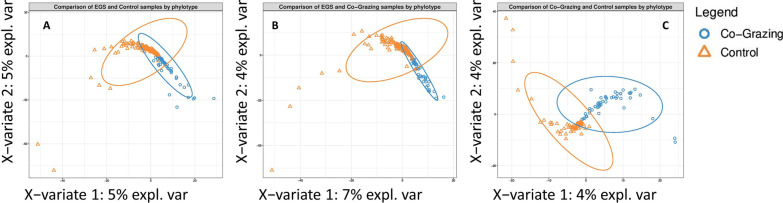
Partial Least Squares Discriminant Analysis (PLS-DA) plots showing separate clustering of all samples from **A** EGS vs CTRL, **B** EGS vs CoG and **C** CoG vs CTRL, at phylotype level. Weighted UniFrac distance analysis identified significant inter-group dissimilarities (Additional file 1; Table S7). Confidence ellipses show 95% confidence level

**Fig. 5:**
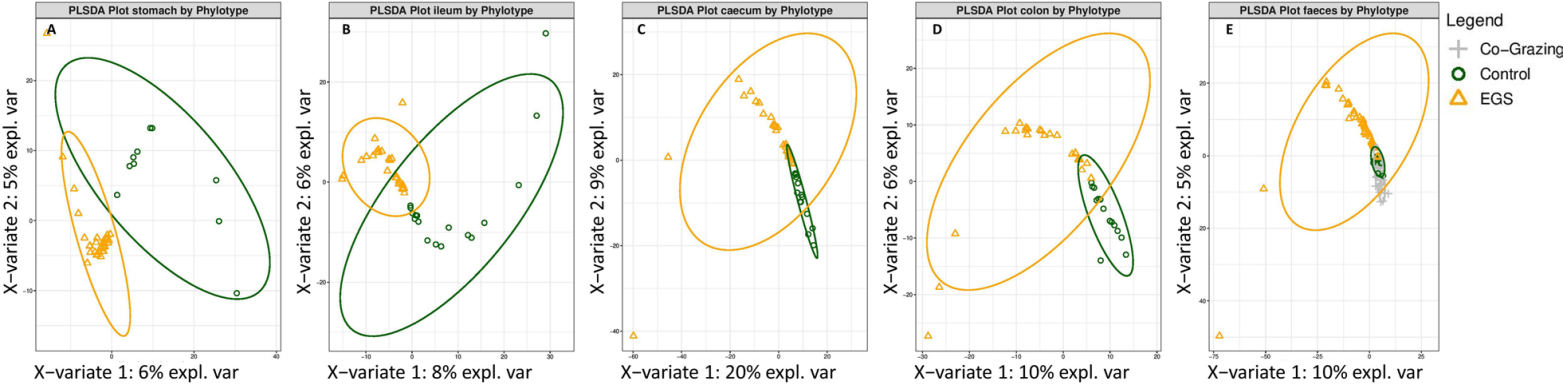
Partial Least Squares Discriminant Analysis (PLS-DA) plots of samples from EGS, CTRL and CoG groups, analysed separately for **A** stomach, **B** ileum, **C** caecum, **D** colon and **E** faeces. Weighted UniFrac distance analysis identified significant inter-group dissimilarities (Additional file 1; Table S7). Confidence ellipses show 95% confidence level

PLS-DA and weighted UniFrac distance analysis (Beta-diversity) identified significant differences in mycobiota structure among GI sites (Fig. 3; Additional file 1; Table S6) and among groups (Fig. 4; Additional file 1; Table S7), at phylotype level. There were significant inter-site dissimilarities in mycobiota structure in EGS horses, between stomach versus colon (*P* = 0.027) and faeces (*P* = 0.0001), and between ileum versus faeces (*P* = 0.01) (Additional file 1; Table S6). Inter-group comparisons of EGS versus CTRL at each GI site identified dissimilarity overall (*P* = 0.023) and in stomach samples (*P* = 0.001) (Figs. 4 and 5; Additional file 1: Table S7). There was also significant dissimilarity in structure of mycobiota in faeces samples from EGS versus CoG (*P* = 0.008) and CoG versus CTRL (*P* = 0.006).

### Identification of differentially abundant phylotypes using inter-group DESeq2 analysis

DESeq2 inter-group analysis identified many differentially abundant phylotypes at Phylum, Class, Order, Family, Genus and phylotype levels (Additional file 1; Table S8 & Additional file 2). Significantly more of the differentially abundant taxa were more abundant in EGS vs CTRL and in CoG vs CTRL groups, at each taxonomic level (paired t test, *P* = 0.001). Phylotypes with increased (*n* = 877) and decreased (*n* = 488) abundance, in at least one GI site, in the EGS group (versus CoG and CTRL groups) and in CoG versus CTRL groups are listed in Additional file 2.

Genera with ≥ 4 phylotypes that had decreased abundance in EGS were *Acremonium*, *Alternaria*, *Aspergillus*, *Candida*, *Cladosporium*, *Fusarium*, *Neoascochyta*, *Penicillium*, *Ramularia*, *Talaromyces*, *Taphrina* (all *Ascomycota*), *Coprinellus*, *Coprinopsis*, *Cutaneotrichosporon*, *Cystofilobasidium*, *Dioszegia*, *Filobasidium*, *Leucosporidium*, *Naganishia*, *Papiliotrema*, *Psathyrella*, *Rhodotorula*, *Udeniomyces*, *Vishniacozyma* (all *Basidiomycota*) and *Mortierella* (*Mortierellomycota*). Of the phylotypes with decreased abundance in EGS horses, the ten with highest variable importance in projection (VIP) scores were *Mycosphaerella tassiana* (VIP 3.20), *Wickerhamomyces anomalus* (2.99), *Filobasidium oeirense* (2.87), *Trichosporon insectorum* (2.83), *Cladosporium grevilleae* (2.77), *Trichosporon lactis* (2.57), *Sporobolomyces roseus* (2.56), G_*Fusarium* (2.47), O_*Hypocreales* (2.41) and *Cladosporium ramotenellum* (2.40) (Additional file 2).

### Key phylotypes associated with EGS

Key phylotypes (*n* = 56; Table 2; Additional file 3) associated with EGS, and which could have a potential role in EGS aetiology, were identified. These had; a) high abundance in EGS samples ie being within the top 50% quantile of abundant phylotypes, b) high prevalence in EGS samples ie present in > 50% of EGS samples included in each paired comparison, c) a VIP score > 1.5 and significantly increased abundance [Padj < 0.05, log2 fold change > 0] in EGS samples in any comparison of EGS versus CTRL (overall, stomach, ileum, caecum, colon, faeces) or EGS versus CoG (faeces) samples. Key phylotypes comprised fungi of diverse taxonomy. All belonged to two phyla, namely *Ascomycota* (*n* = 28) and *Basidiomycota* (*n* = 25), except one from *Mortierellomycota*, one from *Chytridiomycota* and one unidentified at phylum level. Key phylotypes belonged to the ten dominant classes, except for *Neocallimastigomycetes*. Five key phylotypes were unidentified or *incertae sedis* at order level, while the remainder belonged to 24 orders. Fourteen key phylotypes were unidentified or *incertae sedis* at family level, while the remainder belonged to 27 families. Eighteen key phylotypes were unidentified or *incertae sedis* at genus level, while the remainder belonged to 29 genera. Thirty three key phylotypes were identified at species level.

**Table 2:** List of 56 key phylotypes that were associated with EGS

Phylum	Class	Family	Phylotype
*Ascomycota*	*Dothideomycetes*	*Didymellaceae*	*Endophoma_elongata, Phomatodes_aubrietiae*
		*Didymosphaeriaceae*	*Paraconiothyrium_brasiliense*
		*Phaeosphaeriaceae*	G_*Phaeosphaeria*
		*Sporormiaceae*	*Preussia_tetramera*, G_*Preussia*
		Unidentified	O_*Pleosporales*
	*Eurotiomycetes*	*Herpotrichiellaceae*	*Exophiala_pisciphila,* F_*Herpotrichiellaceae*
		*Trichocomaceae*	*Thermomyces_lanuginosus*
		Unidentified	O_*Onygenales*
	*Leotiomycetes*	*Pseudeurotiaceae*	*Pseudeurotium_bakeri, Pseudeurotium_ovale*
		*Thelebolaceae*	*Cleistothelebolus_nipigonensis*
			*Thelebolus_globosus, Thelebolus_spongiae,* G_*Thelebolus*
		Unidentified	O_*Helotiales*
	*Saccharomycetes*	Unidentified	O_*Saccharomycetales*
	*Sordariomycetes*	Unidentified	O_*Coniochaetales*
		*Bionectriaceae*	*Gliomastix_tumulicola*
		*Hypocreaceae*	*Monocillium_griseo-ochraceum*
		*Hypocreales_*fam*_Incertae_sedis*	*Acremonium_rutilum,* G_*Acremonium*
		*Hypocreales_*fam*_Incertae_sedis*	*Chlamydocillium_cyanophilum*
		*Hypocreales_*fam*_Incertae_sedis*	F_*Hypocreales*_fam*_Incertae_sedis*
		*Lasiosphaeriaceae*	F_*Lasiosphaeriaceae*
		*Microascaceae*	*Kernia_retardata*
*Basidiomycota*	*Agaricomycetes*	*Agaricaceae*	*Coprinus_cordisporus*
		*Entolomataceae*	*Entoloma_sericeum*
		*Psathyrellaceae*	G_*Coprinopsis*
		unidentified	O_*Agaricales*
		unidentified	C_*Agaricomycetes*
	*Cystobasidiomycetes*	*Cystobasidiaceae*	*Cystobasidium_minuta, Cystobasidium_ritchiei*
		*Buckleyzymaceae*	*Buckleyzyma_aurantiaca*
		unidentified	O_*Erythrobasidiales*
	*Microbotryomycetes*	*Chrysozymaceae*	*Bannozyma_yamatoana*
			F_*Chrysozymaceae*
		unidentified	O_*Leucosporidiales*
		unidentified	O_*Sporidiobolales*
	*Tremellomycetes*	*Bulleribasidiaceae*	*Vishniacozyma_foliicola*
		*Filobasidiaceae*	*Naganishia_adeliensis, Naganishia_albida*
		*Mrakiaceae*	*Tausonia_pullulans*
		*Piskurozymaceae*	*Solicoccozyma_phenolica*
		*Trimorphomycetaceae*	*Saitozyma_podzolica*
		*Trichosporonaceae*	*Apiotrichum_dulcitum*
		unidentified	C_*Tremellomycetes*
	unidentified	unidentified	P_*Basidiomycota*
	*Wallemiomycetes*	*Wallemiaceae*	*Wallemia_canadensis, Wallemia_sebi, Wallemia_tropicalis*
*Chytridiomycota*	unidentified	unidentified	P_*Chytridiomycota*
*Mortierellomycota*	*Mortierellomycetes*	*Mortierellaceae*	*Mortierella_gamsii*
unidentified	unidentified	unidentified	K_unidentified

Key taxa had significantly increased abundance in EGS samples, variable importance in projection scores > 1.5, and both high abundance and high prevalence in EGS samples. K = kingdom, P = phylum, C = class, O = order, F = family, G = genus

FUNGuild classifications were available for 35 of 56 (63%) key phylotypes. Growth morphologies were predominantly microfungi (37.1%), null (31.4%), yeasts (17.1%) and agaricoids (11.4%)(Additional file 1; Table S2; Additional file 4). Tropic mode was predominantly saprotroph (91.4%), with smaller proportions of pathotrophs (34.3%) and symbiotrophs (28.6%)(Additional file 1; Table S3; Additional file 4). In terms of ecological guild, most phylotypes were assigned to undefined saprotrophs (71.4%), fungal parasites (22.8%), dung saprotrophs (20.0%), endophytes (17.1%), plant pathogens (14.3%), animal pathogens (14.3%) soil saprotrophs (11.4%), wood saprotrophs (11.4%), and plant saprotrophs (8.6%)(Additional file 1; Table S4; Additional file 4).

The structure of the faecal mycobiota of CoG horses differed significantly from those of EGS and CTRL horses (Additional file 2). Of the 56 key phylotypes associated with EGS, 29 were more abundant in faeces of EGS vs CoG groups, while 9 were significantly more abundant in faeces of CoG vs CTRL horses (Additional File 2).

## Discussion

### Grazing horses have a very rich and diverse GI mycobiota

This is the first reported molecular characterisation of the mycobiota throughout the GI tract of grazing horses. All horses, at all 5 GI sites, had a very rich and diverse mycobiota, evidenced by the overall detection of 13,204 OTUs and 2816 phylotypes. The majority of taxa were identified to genus level, but only 40.1% were identified to species level. Dominant phyla, in terms of abundance, were *Ascomycota*, *Basidiomycota* and *Neocallimastigomycota*. Dominant classes were *Dothideomycetes*, *Eurotiomycetes*, *Leotiomycetes*, *Saccharomycetes*, *Sordariomycetes* (Ascomycota), *Tremellomycetes, Wallemiomycetes* (Basidiomycota) and *Neocallimastigomycetes*. Dominant orders were *Capnodiales*, *Eurotiales*, *Pleosporales*, *Saccharomycetales, Thelebolales* (Ascomycota), *Filobasidiales* (Basidiomycota) and *Neocallimastigales*. Dominant families were *Aspergillaceae*, *Phaffomycetaceae, Sporormiaceae*, *Thelebolaceae* (Ascomycota), *Bulleribasidiaceae*, *Filobasidiaceae*, *Wallemiaceae* (Basidomycota) and *Neocallimastigaceae*. Dominant genera were *Aspergillus*, *Preussia*, *Thelebolus*, *Wickerhamomyces* (Ascomycota), *Naganishia*, *Vishniacozyma Wallemia* (Basidiomycota) and *Piromyces* (*Neocallimastigomycota*). Dominant species were *Aspergillus proliferans*, *Wickerhamomyces anomalus* (Ascomycota) and *Vishniacozyma victoriae*, *Wallemia muriae, W. sebi* (Basidiomycota).

FUNGuild analysis parsed 87% of the 2816 phylotypes into 20 growth morphologies, 26 ecological guilds and 3 tropic modes. Most were classified as environmental microfungi, agaricoids or yeasts, comprising wood, soil, plant, dung and undefined saprotrophs, plant pathogens, endophytes, animal pathogens, fungal parasites and ectomycorrhizal fungi. These fungi are typical of those colonising grassland soils and plants [33] and show considerable overlap with those identified in a metagenomic study of grass endophytes [34].

The GI mycobiota of grazing horses appears to be richer and more diverse than that of humans and mice, which typically comprise nearly 70 genera and more than 184 species of fungi, but with 10 or fewer taxa typically accounting for the vast majority of fungi detected [35–38]. The marked richness and diversity of the equine GI mycobiota likely reflects the richness and diversity of the environmental fungi present in the plants, soil and water that are ingested by grazing horses. Consistent with this, the diversity of fungal species in equine faeces was considered to reflect the different forage types fed to stabled horses [39]. Similarly, most of the fungi detected in human faeces are derived from the consumption of different foods, which contain, as a whole, more unique fungi than the population colonising the GI tract [40]. Many of the fungi commonly considered to represent the human core GI mycobiota, including *Candida*, *Saccharomyces*, *Penicillium*, *Aspergillus*, *Cryptococcus*, *Malassezia*, *Cladosporium* and *Trichosporon* [36, 41, 42], were detected in the equine GI tract. The predominant fungal phyla in both equine and human GI tracts are *Ascomycota* and *Basidiomycota*, while *Neocallimastigomycota,* GI adapted anaerobic fibre degrading endosymbionts, are abundant only in the horse [43, 44]. *Neocallimastigomycota* are an essential part of the core mycobiota colonising the equine GI tract, but the composition of the remainder of the equine core GI mycobiota is unknown and cannot be determined from this study alone. Indeed it is currently unclear whether the aerobic fungi detected in the GI tract of horses and other animals, including man, are true endosymbionts, opportunistic pathogens which colonise the GI tract only under particular circumstances, or are ingested fungi from food, water, environment, and nasal or oral cavities, and which are simply transiting through the GI tract [36, 42]. While the majority of aerobic fungi detected in the equine GI tract are likely to be transient non-colonising ingested environmental fungi, because these can maintain metabolic activity during GI transit [45], they could potentially contribute to EGS aetiology by producing extrolites in vivo. Opportunistic fungal pathogens which have colonised the equine GI tract could also contribute to EGS aetiology by producing toxic extrolites in vivo.

Mycobiota richness (Chao1) varied throughout the equine GI tract, being higher distally (caecum, colon, faeces) than proximally (stomach, ileum). PLS-DA and weighted UniFrac distance analysis (beta-diversity) identified significant differences in mycobiota structure among GI sites in both EGS and CTRL groups. *Neocallimastigomycota, Neocallimastigomycetes*, *Neocallimastigales* and *Neocallimastigaceae* were more abundant in distal than proximal GI sites, consistent with previous findings [46]. In contrast, *Wallemiomycetes*, *Wallemiales*, *Wallemiaceae* and *Tremellales* were more abundant proximally.

### EGS is associated with changes in the richness, diversity and structure of the GI mycobiota

EGS is associated with significant alterations in the GI mycobiota. Mycobiota richness (Chao1) was higher in the caecum and colon of EGS horses compared with CTRL horses, while mycobiota diversity (Inverse Simpson) was higher in EGS colon and faeces, compared with CTRL and CoG horses, respectively. Indices of beta-diversity demonstrated inter-group differences in mycobiota structure at all taxonomic levels. Analysis with the Bioconductor software package DESEq2 identified a large number of phylotypes that were differentially abundant between EGS and the 2 control groups, most of which had increased abundance in EGS horses. PLS-DA and VIP scores (> 1.5) were used to identify those taxa that were the most important contributors to the inter-group differential mycobiota structure. Key phylotypes (*n* = 56) associated with EGS, and which could have a potential role in EGS aetiology, were then identified which had a) high abundance and high prevalence in EGS samples, b) significantly increased abundance in EGS samples, and c) a VIP score > 1.5 indicating they contributed significantly to inter-group differences in mycobiota structure. Key phylotypes comprised fungi of diverse taxonomy. FUNGuild analysis parsed the key phylotypes as predominantly environmental microfungi, classified as soil, dung, wood, plant and undefined saprotrophs, fungal parasites, plant pathogens, endophytes and animal pathogens. Some key phylotypes were macrofungi; *Entoloma sericeum*, G*_Coprinopsis*, O*_Agaricales* and C*_Agaricomycetes*.

The increased abundance of key phylotypes in the GI tract of EGS horses could reflect increased GI colonisation by opportunistic pathogenic fungi, but more likely reflects ingestion of increased numbers of these fungi in plants, litter and soil while grazing. Indeed there is evidence to suggest that EGS horses are exposed to increased numbers of a wide range of diverse environmental microbes. In addition to key fungi, EGS horses had increased abundance of K_*Rhizaria* (G_*Cercozoa*), heterotrophic protists that predate bacteria thereby substantially changing the structure and function of microbial communities on plant surfaces [47–49]. Further, a previous study identified increased numbers of cyanobacteria, filamentous green algae, unicellular green algae, diatoms, motile algal flagellates and desmids (*Closterium* sp.) on plants growing on EGS pastures during disease outbreaks [50]. It is likely that this increased abundance of a wide range of diverse microbes on EGS fields reflects favourable environmental conditions for microbial growth, including suitable vegetation, soil organic matter content, pH, conductivity, temperature and availability of water and macronutrients [51]. Conditions which favour fungal growth and/or extrolite elaboration on the pasture could potentially account for some of the environmental risk factors for EGS. These factors include spring and early summer season, cool, dry weather with irregular ground frosts, faecal contamination, sand and loam rather than chalk soils, high soil nitrogen and low Cu and Zn, and pasture disturbance [15, 20, 52]. Many key phylotypes are extremophilic fungi, including *Pseudeurotium*, *Thelobolus* (psychrophilic), *Thermomyces lanuginosus* (thermophilic), *Wallemia* (xerophilic) and extremophilic or polyextremophilic yeasts including *Apiotrichum*, *Bannozyma, Cystobasidium*, *Nagashinia*, *O_Saccharomycetales*, *Saitozyma*, *Tausonia* and *Vishniacozyma* [51, 53, 54]. Increased abundance of these extremophiles likely reflects their ability to survive the potentially adverse environmental conditions associated with EGS, including cold and dry weather. The increased abundance of some soil yeasts may also be attributable to extracellular polysaccharide capsules that facilitate survival in sandy soils [51] which are associated with EGS [20]. Many key phylotypes are dung saprotrophs, including* Acremonium* spp. O_*Agaricales, Coprinus* spp., *Coprinopsis* spp.,* Pleospora* spp.,* Preussia* spp.,* Thelebolus* spp. [55], *Cleistothelebolus nipigonensis*, O*_Coniochaetales* and *Kernia retardata*, potentially explaining the reduction in EGS risk when faeces are collected manually from pastures [20].

Alternatively, the alterations in mycobiota associated with EGS could be a consequence, rather than a cause of EGS, perhaps attributable to the GI stasis which characterises the disease. Consistent with this possibility, experimental murine antimycotic drug-induced intestinal fungal dysbiosis resulted in increased abundance of *Wallemia* [56], one of the key phylotypes associated with EGS. Further work is therefore required to determine whether any of the key phylotypes contribute to EGS aetiology or whether their association with EGS is a consequence of the disease. None of the key phylotypes has been previously associated with a pasture neuromycotoxicosis resembling EGS, however many of them are predicted to produce cytotoxic and/or neurotoxic extrolites, including brefeldin, communiols, cytochalasans, d-lysergic acid amide, gliotoxin, L-DOPA, polyketides, preussins, sesquiterpenoids, trichothecenes, tyrosinase and walleminol [39, 57–59]. In addition, antibacterial and antifungal activities of fungal extrolites within the GI tract [60] could potentially induce the marked GI bacterial dysbiosis which occurs in EGS [61] and contribute to the changes in mycobiota reported herein. Examination of GI contents from EGS horses for those extrolites produced by key phylotypes is therefore warranted to further test the hypothesis that they contribute to EGS aetiology.

It is conceivable that EGS is more prevalent in horses when there is a reduced abundance of particular taxa that serve key beneficial functions for the host. Notable taxa with reduced abundance in EGS horses included some species of *Alternaria* and *Cladosporium*, dominant endophytic fungi on temperate grasses [62], *Fusarium*, an animal and plant pathogen, soil and wood saprotroph, endophyte, and lichen parasite, *Neoascochyta*, an animal and plant pathogen and saprotroph, and the plant pathogen *Mycosphaerella* *tassiana.* Consistent with these findings, Doxey et al. [22] isolated *Fusarium* and *Alternaria* only infrequently from EGS horses. In contrast, Robb et al. [24] identified *Fusarium* on plants from all EGS fields in Scotland and Patagonia. Rather than contributing to EGS aetiology, reduced numbers of these aforementioned taxa likely reflects reductions in the numbers of these fungi that are ingested by EGS horses, perhaps because the environmental conditions associated with EGS are unfavourable for growth of these fungi. Alternatively, reduced numbers of certain taxa may be attributable to the inhibitory effects of antifungal extrolites produced by those taxa that were present in increased abundance.

The structure of the faecal mycobiota of CoG horses differed significantly from those of EGS and CTRL horses. CoG horses were co-grazing with EGS horses at the time of disease onset. While CoG horses remain clinically healthy, as with EGS horses, they have increased serum concentrations of acute phase proteins [32] consistent with subclinical exposure to the toxin that causes EGS. It is possible that the quantity of causal fungi/extrolite ingested by CoG horses is sufficient to induce a self-resolving acute inflammatory response but insufficient to induce clinical EGS. Consistent with this possibility, 29 of the 56 key phylotypes associated with EGS were more abundant in faeces of EGS versus CoG groups. Alternatively CoG horses may survive exposure to the causal toxin because of host-specific immunological protection or toxin metabolism.

### Study limitations

Targeted amplicon sequencing identified considerably many more fungi than a previous culture-based study of the GI mycobiota of EGS and control horses [22]. However, this methodology has biases and limitations [43], including underestimation, or failure to detect, taxa lacking validated phylogenetic marker sequences, such as some grass endophytes [62] and *Fusarium* [63]. This may also explain why several taxa isolated from the equine GI tract by Doxey et al. [22] were not identified in the present study, including *Absidia*, *Rhizopus*, *Thamnidium*, *Geotrichum (Dipodascus)* and *Monilia*. The limited annotation of some fungal genes can also preclude classification of taxa to fine taxonomic ranks. In the present study, most taxa were identified to genus level, but only 40.1% were identified to species level, and some taxa, including key taxa, were identified only at kingdom or phylum levels.

While all of the fungi potentially present in the equine GI tract of grazing horses will not have been identified in this study, this was not considered to be a significant limitation given that the main aim of the study was to identify key taxa associated with EGS, rather than to generate an inventory of the equine GI mycobiota.

Inclusion of mock community and negative controls is considered an essential feature of amplicon sequencing experiments, to identify biases and sample contamination, respectively [63]. All 12 taxa in the two mock communities were identified in all mock community samples. Samples of mock community 1 had markedly lower amplicon numbers for *Saccharomyces cerevisiae* than for *Cryptococcus neoformans*. Similarly, Bakker [63] failed to detect *S. cerevisiae* in an ITS1 amplicon library, concluding that this negative bias was likely associated with amplicon length. We have noted that the number and depth of OTUs is immensely sensitive to the stringency of the parameters applied during assembly (data not shown), and thus the abundance of rRNA gene amplicons may not accurately reflect taxon biomass in samples [63]. Some taxa, including *Coniochaeta lignicola* which was included in mock community 2, were represented by a single OTU. Others, including *Vishniacozyma victoriae,* were represented by multiple OTUs, reflecting intragenomic marker gene polymorphism [64]. To mitigate against these observations, a phylotype approach, rather than an OTU approach, was adopted for this study [63].

Negative control samples are valuable for revealing rDNA contamination, but there is little or no consensus regarding how to incorporate information from negative control samples into data processing [63]. All negative control samples had low amplicon counts, except 3 which were contaminated with rDNA from *Alternaria infectoria* and *Mycosphaerella tassiana*. As these two taxa were included in mock community 2, and were present in many GI samples, it was considered inappropriate to remove these taxa from the GI sample analysis [63]. Importantly, these contaminant taxa were not key taxa and were not increased in abundance in EGS samples. Retrospective analysis indicated that 53 of the 13,204 OTUs were likely contaminants; none of these contributed to the key phylotypes.

EGS horses were significantly younger than controls, in part reflecting the difficulty obtaining post-mortem samples from young control horses. While age influences mycobiota structure [65], it seems unlikely to account for the significant inter-group differences observed herein, which are instead consistent with inter-group differences in exposure to a diverse range of environmental fungi.

## Conclusions

The equine GI mycobiota comprises a very large and diverse population of fungi, varying in growth form, trophic mode and ecological guild. Most are ingested environmental fungi, probably in transit through the GI tract. EGS horses had a significantly richer, more diverse, and structurally different, GI mycobiota than two control populations. A large number of taxa showed statistically significant differential abundances between groups. Key phylotypes (*n* = 56) associated with EGS were identified, many of which are extremophiles capable of producing cytotoxic and/or neurotoxic extrolites. Further work is required to determine whether extrolites produced by key phylotypes contribute to EGS aetiology or whether the association of key phylotypes and EGS is non-causal or is a consequence of the disease.

## Methods

### Sample collection and processing

GI contents were collected *post mortem*, typically within 4 h of euthanasia on humane grounds by barbiturate overdose, from up to 5 sites, namely stomach, ileum, caecum, colon and rectum (faeces) of EGS horses (150 samples from 54 horses) and from control grazing horses that had been euthanased on humane grounds for reasons other than neurologic or GI diseases (CTRL group; 67 samples from 31 horses) (Additional file 5). We were unable to sample all GI sites in all horses. EGS horses comprised 39 acute EGS and 15 sub-acute EGS horses, categorised as previously described [25], and confirmed by histopathology of autonomic ganglia. Freshly voided faecal samples were also collected from 48 healthy horses that were co-grazing (CoG group) with the EGS horses when the latter developed EGS, typically within 48 h of disease onset. Samples were promptly frozen at − 20 or − 80 °C. In addition to the 265 GI samples described, an additional 26 samples were collected and processed, but were not included in the subsequent analyses because they generated too few (< 5000) amplicon sequences.

Negative (nuclease-free water) and positive mock fungal community controls were run in parallel with samples. Initial samples were run in parallel with mock community 1 (10 ng DNA per sample), a mixed microbial population which included rDNA from *Saccharomyces cerevisiae* and *Cryptococcus neoformans* (ZymoBIOMICS Microbial Community DNA Standard, Zymo Research, Irvine, USA). Subsequent samples were run with mock community 2, which was prepared to provide a community of 10 fungi (*Alternaria infectoria*, *Coniochaeta lignicola*, *Didymella rumicicola, Mycosphaerella tassiana, Penicillium pagulum, Pyrenochaetopsis pratorum*, *Vishniacozyma victoriae*, *Xylaria longipes,* G_*Eutypella and* G_*Fusarium*) which colonised grasses collected from equine pastures within the geographical area from which the equine GI samples were collected. To prepare mock community 2, plant stems and crowns were surface sterilised [66] and cultured on corn meal agar at room temperature. On day 10, 33 isolates were selected and sub-cultured and then curated on day 28. Mycelia were harvested from 10 selected isolates, homogenised mechanically and rDNA extracted using a standard CTAB DNA extraction protocol. DNA was quantified using NanoDrop 2000 spectrophotometer (NanoDrop Technologies, Wilmington, DE, USA), and quantity assessed using Qubit 3 (Invitrogen, Inchinnan, UK). Mock community 2A contained 5 ng DNA from all 10 isolates, while mock community 2B contained 50 ng DNA of *Didymella rumicicola* and *Xylaria longipes* and 5 ng for other isolates.

For GI samples, total DNA was extracted with the AllPrep PowerFecal DNA/RNA Kit (QIAGEN, Hilden, Germany), following manufacturer’s instructions. A bead beating step was included (FastPrep-24 5G bead beating grinder and lysis system, MP Biomedicals, Eschwege, Germany). The purity of the resulting DNA extract was assessed using NanoDrop 2000 spectrophotometer (NanoDrop Technologies, Wilmington, DE, USA), and the quantity assessed using Qubit 3 (Invitrogen, Inchinnan, UK) and the Agilent High Sensitivity D1000 ScreenTape System (2200)(Agilent Technologies, Santa Clara, USA). Pooled libraries also underwent quality control checks by Edinburgh Genomics [67] prior to sequencing using the High Sensitivity D1K ScreenTape (Agilent Technologies, Santa Clara, USA).

The Internal Transcribed Spacer 1 (ITS1) region was amplified using primers ITS1F (FWD 5`-CTTGGTCATTTAGAGGAAGTAA-3`) and ITS2 (REV 5`- GCTGCGTTCTTCATCGATGC -3`) [68], using 50 ng DNA per sample. Amplification was performed on a Mycycler Thermal Cycler (Bio-Rad, Watford, UK). PCR cycle conditions, selected based on primer recommendations, were 95 °C for 2 min, 95 °C for 30 s, 55 °C for 30 s and 72 °C for 1 min (× 30 cycles) and 72 °C for 7 min. DNA amplicons were purified using the AMPure XP PCR Purification System (Beckman Coulter, High Wycombe, UK) according to manufacturer’s instructions. Amplicons were paired-end (2 × 250 nt) sequenced on Illumina MISeq v2 platform (Illumina, San Diego, USA).

### Bioinformatics and statistical analyses

Microbial composition summary box plots, alpha diversity, weighted UniFrac based Partial Least-Squares Discriminant Analysis (PLS-DA) were performed within R version 3.6.0 (2019–04-26) [69] using the following packages: phyloseq [70], metagenomeSeq [71], vegan [72], ape [73], ggplot2 [74], mixOmics [75], DESeq2 [76], GUniFrac [77] and dplyr [78]. A paired t-test on the sample count for each month was used to determine whether there was an inter-group difference in month of collection of EGS and CTRL samples. Chi squared test was used to determine whether there were inter-group differences in horse sex. Mann Whitney tests were used to determine whether there were inter-group differences in horse age.

Raw sequence data from 3 MiSeq runs were pooled and analysed with PIPITS [79]*,* an open‐source (https://sourceforge.net/projects/pipits) stand‐alone suite of software for automated processing of Illumina MiSeq sequences for fungal community analysis, incorporating ITSx to extract subregions of ITS and exploiting the latest RDP Classifier to classify sequences against the curated UNITE fungal ITS reference data set [80]. GI samples (*n* = 26 from all groups) with total amplicon read counts < 5000 were not included in the study, leaving 265 GI samples for analysis. Sequencing depth was assessed using rarefaction curves. Taxonomy plots were constructed for phylum, class, order and family, using data normalised to relative abundance. To aid visualisation, plots were constructed using data filtered at 0.05% abundance. Data were analysed using the R package decontam [81]; since this was done retrospectively, identified contaminants were not removed and were included in the analyses.

FUNGuild [33] was used to taxonomically parse phylotypes to defined ecological guilds, trophic modes and growth morphologies. Alpha-diversity was measured using Chao1 (richness estimator that accounts for sequencing depth) and Inverted Simpson (diversity). Alpha-diversity indices were calculated at phylotype level using the “Phyloseq” R package [70]. Kruskal–Wallis rank sum test and Wilcoxon rank sum test with continuity correction were used to determine whether indices differed among GI sites and among groups.

Partial least squares discriminant analysis (PLS-DA), a supervised model based on a least squares regression model, was used to reveal inter-group and inter-site variation in mycobiota structure at phylotype level. Differences were assessed by performing an adonis analysis based on weighted UniFrac distances, a phylogenetic based distance metric which, when weighted, accounts for the relative abundance of phylotypes [82]. DESeq2 analysis was used for inter-group comparisons (EGS versus CTRL, EGS versus CoG and CoG versus CTRL) of mycobiota, for each GI site separately, at all taxonomic levels except kingdom, at phylotype level. PLS-DA and variable importance in projection (VIP) scores were used to identify phylotypes that were important contributors to the differential mycobiota structure in EGS versus CTRL groups, EGS versus CoG groups and CoG versus CTRL groups, using the “plsda” function in the R package “mix Omics” [75, 83]. Phylotypes with VIP scores > 1.5 were considered to be important contributors to the model [84]. Key phylotypes associated with EGS, and which could have a potential role in EGS aetiology, were identified that had; a) high abundance in EGS samples ie being within the top 50% quantile of abundant phylotypes, b) high prevalence in EGS samples ie present in > 50% of EGS samples included in each paired comparison, c) a VIP score > 1.5 and significantly increased abundance [Padj < 0.05, log2 fold change > 0] in EGS samples in any comparison of EGS versus CTRL (overall, stomach, ileum, caecum, colon, faeces) or EGS versus CoG (faeces) samples. For this, DESeq2 and PLS-DA/VIP analyses were performed including only those phylotypes which had the aforementioned high prevalence and abundance in EGS samples.

## Supplementary Information

**Additional file 1: Table S1**. Numbers of OTUs, phylotypes and high quality sequences in equine GI samples (*n* = 265) from EGS (*n* = 31), CTRL (*n* = 54) and CoG (*n* = 48) horses. **Figure S1.** Rarefaction curve of phylotype richness for individual GI samples (*n* = 265) showing adequate coverage. **Table S2.** Number and % of phylotypes (assignments were available for 2460/2816 phylotypes) and key phylotypes (assignments were available for 35/56 key phylotypes) assigned to each of the 20 growth morphologies. Some phylotypes and key phylotypes were assigned to multiple growth morphologies. **Table S3.** Number and % of phylotypes (assignments were available for 2460/2816 phylotypes) and key phylotypes (assignments were available for 35/56 key phylotypes) assigned to each of the 3 FUNGuild trophic modes. Some phylotypes and key phylotypes were assigned to multiple trophic modes. **Table S4.** Number and % of phylotypes (assignments were available for 2460/2816 phylotypes) and key phylotypes (assignments were available for 35/56 key phylotypes) assigned to each of 26 ecological guilds. Some phylotypes and key phylotypes were assigned to multiple ecological guilds. **Figure S2.** Taxonomy plots showing relative abundance of taxa at (A) order and (B) family levels. Data are filtered at 0.05% abundance threshold. **Table S5.** Statistical comparison of indices of alpha-diversity across all 5 GI sites (overall) and between paired GI sites. Data pooled for all horses; ANOVA, P values. Significant differences in bold, letter indicates higher value (Ca = caecum; F = faeces). **Table S6.** Weighted UniFrac distance analysis identified significant inter-site dissimilarity in mycobiota structure in EGS and CTRL horses, at phylotype level. P values, statistically different comparisons are indicated in bold. **Table S7.** Inter-group weighted UniFrac distance analysis at different GI sites, at phylotype level (P values). Statistically significant dissimilarity is indicated in bold. **Table S8.** Numbers of differentially abundant taxa, at phylum, class, order, family, genus and phylotype levels.

**Additional file 2**. Differentially abundant (increased and decreased) taxa. Separate sheets show differentially abundant taxa at Phylum, Class, Order, Family, Genus, phylotype (increased and decreased abundance) and OTU (increased and decreased abundance) levels. Separate columns show statistical data for comparisons overall (OV; pooled data for all GI sites) and at paired GI sites (ST; stomach, IL; ileum, CA; caecum, CO; colon, FA; faeces), for EGS vs CTRL, and for faeces for EGS vs CoG and for CoG vs CTRL groups. Separate columns show adjusted p value (padj) and log_2_ fold change, and for phylotype data, VIP scores for statistically significant paired comparisons.

**Additional file 3**. List of key phylotypes associated with EGS (n = 56). Separate columns show taxonomy of key phylotypes and statistical data for comparisons overall (OV; pooled data for all GI sites) and at paired GI sites (ST; stomach, IL; ileum, CA; caecum, CO; colon, FA; faeces), for EGS vs CTRL, and for faeces for EGS vs CoG and for CoG vs CTRL groups. Separate columns show adjusted p value (padj) and log_2_ fold change, and for phylotype data, VIP scores for statistically significant paired comparisons.

**Additional file 4**. FUNGuild parsing of key phylotypes. Separate columns show taxonomy of key phylotypes and their FUNGuild identity, taxon level, trophic mode, guild, growth form, trait, notes, confidence rank, and citation/source, as described by Nguyen et al. (2016) [33] and https://github.com/UMNFuN/FUNGuild.

**Additional file 5**. Horse metadata. Separate columns show sample number, case identity, group, category, GI site, collection date, month of sample collection, premises code (anonymised), age (years), sex and breed. AGS acute grass sickness; SAGS sub-acute grass sickness; ID Irish Draught; TB Thoroughbred; WB Warmblood; x crossbred.

## Data Availability

Sequencing reads can be accessed in the European Nucleotide Archive under accession number PRJEB45209.

## Abbreviations

CoG: Co-grazing group
CTRL: Control group
EGS: Equine grass sickness
GI: Gastrointestinal
ITS: Internal transcribed spacer
OTU: Operational taxonomic unit
PCoA: Principal coordinate analysis
PCR: Polymerase chain reaction;
PLS-DA: Partial least squares discriminant analysis
VIP: Variable importance in projection
