# Supplementary material for: Equine grass sickness (a multiple systems neuropathy) is associated with alterations in the gastrointestinal mycobiome
Source: Anim Microbiome. 2021 Oct 9;3:70. doi: 10.1186/s42523-021-00131-2 (PMC8501654; doi:10.1186/s42523-021-00131-2)
Supplement: Supplementary file 1 — Additional file 1: Table S1. Numbers of OTUs, phylotypes and high quality sequences in equine GI samples (n = 265) from EGS (n = 31), CTRL (n = 54) and CoG (n = 48) horses. Figure S1. Rarefaction curve of phylotype richness for individual GI samples (n = 265) showing adequate coverage. Table S2. Number and % of phylotypes (assignments were available for 2460/2816 phylotypes) and key phylotypes (assignments were available for 35/56 key phylotypes) assigned to each of the 20 growth morphologies. Some phylotypes and key phylotypes were assigned to multiple growth morphologies. Table S3. Number and % of phylotypes (assignments were available for 2460/2816 phylotypes) and key phylotypes (assignments were available for 35/56 key phylotypes) assigned to each of the 3 FUNGuild trophic modes. Some phylotypes and key phylotypes were assigned to multiple trophic modes. Table S4. Number and % of phylotypes (assignments were available for 2460/2816 phylotypes) and key phylotypes (assignments were available for 35/56 key phylotypes) assigned to each of 26 ecological guilds. Some phylotypes and key phylotypes were assigned to multiple ecological guilds. Figure S2. Taxonomy plots showing relative abundance of taxa at (A) order and (B) family levels. Data are filtered at 0.05% abundance threshold. Table S5. Statistical comparison of indices of alpha-diversity across all 5 GI sites (overall) and between paired GI sites. Data pooled for all horses; ANOVA, P values. Significant differences in bold, letter indicates higher value (Ca = caecum; F = faeces). Table S6. Weighted UniFrac distance analysis identified significant inter-site dissimilarity in mycobiota structure in EGS and CTRL horses, at phylotype level. P values, statistically different comparisons are indicated in bold. Table S7. Inter-group weighted UniFrac distance analysis at different GI sites, at phylotype level (P values). Statistically significant dissimilarity is indicated in bold. Table S8. Numbers of differentially a [file 42523_2021_131_MOESM1_ESM.docx]

**Additional file 1**

**Table S1; Numbers of OTUs, phylotypes and high quality sequences in equine GI samples (*n=*265) from EGS (*n=*31), CTRL (*n=*54) and CoG (*n=*48) horses.**

|  |  | **n** | **OTUs** | **Phylotypes** | **Sequences** |
| --- | --- | --- | --- | --- | --- |
| **CTRL** | **Stomach** | 10 | 3416 | 979 | 98,8105 |
|  | **Ileum** | 17 | 4280 | 1168 | 1,602,418 |
|  | **Caecum** | 13 | 3388 | 803 | 673,960 |
|  | **Colon** | 14 | 3267 | 763 | 1,003,297 |
|  | **Faeces** | 13 | 3700 | 952 | 953,890 |
| **EGS** | **Stomach** | 32 | 5842 | 1448 | 2,530,483 |
|  | **Ileum** | 26 | 5371 | 1294 | 1,243,588 |
|  | **Caecum** | 29 | 8038 | 1908 | 2,536,764 |
|  | **Colon** | 24 | 6519 | 1527 | 1,747,884 |
|  | **Faeces** | 39 | 8033 | 1827 | 3,643,478 |
| **CoG** | **Faeces** | 48 | 6700 | 1617 | 6,666,542 |
| **TOTAL** |  | **265** | **13,204** | **2816** | **23,590,409** |

**Figure S1.** Rarefaction curve of phylotype richness for individual GI samples (*n=*265) showing adequate coverage.


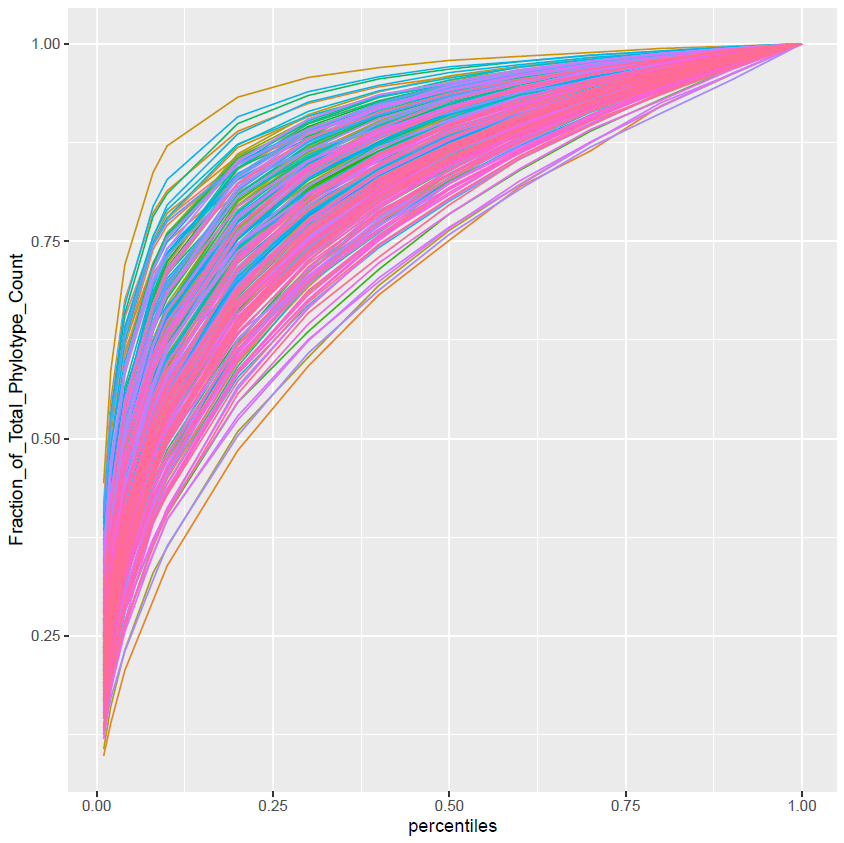


**Table S2; Number and % of phylotypes (assignments were available for 2460/2816 phylotypes) and key phylotypes (assignments were available for 35/56 key phylotypes) assigned to each of the 20 FUNGuild growth morphologies. Some phylotypes and key phylotypes were assigned to multiple growth morphologies.**

| Growth morphology | Number of phylotypes in entire database | % of phylotypes in entire database | Number of key phylotypes | % of key phylotypes |
| --- | --- | --- | --- | --- |
| Microfungus | 803 | 32.7 | 13 | 37.1 |
| NULL | 743 | 30.2 | 11 | 31.4 |
| Agaricoid | 361 | 14.7 | 4 | 11.4 |
| Yeast | 234 | 9.5 | 6 | 17.1 |
| Tremelloid | 91 | 3.7 | 2 | 5.7 |
| Facultative yeast | 90 | 3.7 | 3 | 8.6 |
| Corticioid | 77 | 3.1 |  |  |
| Thallus | 64 | 2.6 |  |  |
| Gasteroid | 57 | 2.3 | 2 | 5.7 |
| Polyporoid | 30 | 1.2 |  |  |
| Secotioid | 25 | 1.0 | 2 | 5.7 |
| Clavarioid | 18 | 0.7 |  |  |
| Dark Septate Endophyte | 16 | 0.7 |  |  |
| Pezizoid | 14 | 0.6 |  |  |
| Dark Septate Microfungus | 10 | 0.4 |  |  |
| Boletoid | 9 | 0.4 |  |  |
| Xylarioid | 3 | 0.1 |  |  |
| Phalloid | 2 | 0.1 |  |  |
| Cordyceptoid | 2 | 0.1 |  |  |
| Hydnoid | 1 | 0.0 |  |  |

**Table S3; Number and % of phylotypes (assignments were available for 2460/2816 phylotypes) and key phylotypes (assignments were available for 35/56 key phylotypes) assigned to each of the 3 FUNGuild trophic modes. Some phylotypes and key phylotypes were assigned to multiple trophic modes.**

| Trophic Mode | Number of phylotypes in entire database | % of phylotypes in entire database | Number of key phylotypes | % of key phylotypes |
| --- | --- | --- | --- | --- |
| Saprotroph | 1849 | 75.3 | 32 | 91.4 |
| Pathotroph | 860 | 35.0 | 12 | 34.3 |
| Symbiotroph | 637 | 25.9 | 10 | 28.6 |

**Table S4; Number and % of phylotypes (assignments were available for 2460/2816 phylotypes) and key phylotypes (assignments were available for 35/56 key phylotypes) assigned to each of 26 ecological guilds.** **Some phylotypes and key phylotypes were assigned to multiple ecological guilds.**

| Guild | Number of phylotypes in entire database | % of phylotypes in entire database | Number of key phylotypes | % of key phylotypes |
| --- | --- | --- | --- | --- |
| Undefined Saprotroph | 1354 | 55.1 | 25 | 71.4 |
| Plant Pathogen | 570 | 23.2 | 5 | 14.3 |
| Wood Saprotroph | 410 | 16.7 | 4 | 11.4 |
| Endophyte | 314 | 12.8 | 6 | 17.1 |
| Animal Pathogen | 291 | 11.8 | 5 | 14.3 |
| Fungal Parasite | 252 | 10.3 | 8 | 22.8 |
| Ectomycorrhizal | 218 | 8.9 | 2 | 5.7 |
| Soil Saprotroph | 183 | 7.4 | 4 | 11.4 |
| Dung Saprotroph | 168 | 6.8 | 7 | 20.0 |
| Plant Saprotroph | 137 | 5.6 | 3 | 8.6 |
| Lichen Parasite | 81 | 3.3 | 0 | 0.0 |
| Litter Saprotroph | 81 | 3.3 | 1 | 2.9 |
| Lichenized | 69 | 2.8 | 0 | 0.0 |
| Leaf Saprotroph | 42 | 1.7 | 1 | 2.9 |
| Bryophyte Parasite | 36 | 1.5 | 1 | 2.9 |
| Epiphyte | 32 | 1.3 | 0 | 0.0 |
| Ericoid Mycorrhizal | 30 | 1.2 | 0 | 0.0 |
| Animal Endosymbiont | 24 | 1.0 | 0 | 0.0 |
| Clavicipitaceous Endophyte | 16 | 0.7 | 0 | 0.0 |
| NULL | 10 | 0.4 | 0 | 0.0 |
| Plant parasite | 9 | 0.4 | 1 | 2.9 |
| Arbuscular Mycorrhizal | 6 | 0.2 | 0 | 0.0 |
| Algal Parasite | 4 | 0.2 | 0 | 0.0 |
| Orchid Mycorrhizal | 4 | 0.2 | 0 | 0.0 |
| Animal Parasite | 2 | 0.1 | 0 | 0.0 |
| Undefined Symbiotroph | 1 | 0.0 | 0 | 0.0 |

**Figure S2: Taxonomy plots showing relative abundance of taxa at (A) order and (B) family levels. Data are filtered at 0.05% abundance threshold.**


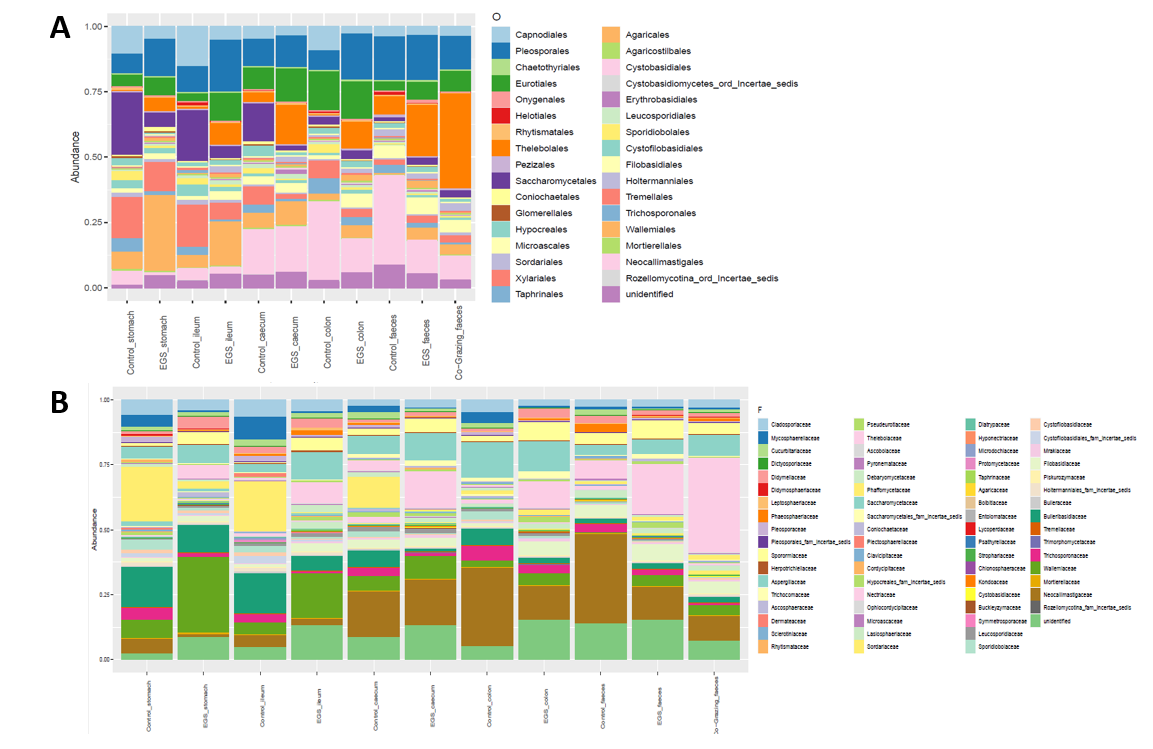


**Table S5; Statistical comparison of indices of alpha-diversity across all 5 GI sites (overall) and between paired GI sites. Data pooled for all horses; P values; significant differences in bold, letter indicates higher value (Ca=caecum; F=faeces).**

|  | Overall |  | Stomach | Ileum | Caecum | Colon |
| --- | --- | --- | --- | --- | --- | --- |
| Chao1 | **0.0084** | Ileum | 0.906 |  |  |  |
|  |  | Caecum | 0.0727 | 0.0857 |  |  |
|  |  | Colon | 0.3646 | 0.3292 | 0.5881 |  |
|  |  | Faeces | **0.00364 (F)** | **0.001898 (F)** | 0.4622 | 0.1676 |
| Inverse Simpson | 0.1914 | Not done since no significant difference overall | | | | |

**Table S6; Weighted UniFrac distance analysis identified significant inter-site dissimilarity in mycobiota structure in EGS and CTRL horses, at phylotype level. P values, statistically different comparisons in bold.**

|  |  | Stomach | Ileum | Caecum | Colon |
| --- | --- | --- | --- | --- | --- |
| EGS | Ileum | 0.721 |  |  |  |
|  | Caecum | 0.1 | 0.652 |  |  |
|  | Colon | **0.027** | 0.247 | 0.826 |  |
|  | Faeces | **0.001** | **0.01** | 0.75 | 0.394 |
| CTRL | Ileum | 0.996 |  |  |  |
|  | Caecum | 0.572 | 0.794 |  |  |
|  | Colon | 0.244 | 0.344 | 0.924 |  |
|  | Faeces | 0.334 | 0.138 | 0.407 | 0.793 |

**Table S7; Inter-group weighted UniFrac distance analysis at different GI sites, at phylotype level (P values). Statistically significant dissimilarity is indicated in bold.**

|  |  | Phylotypes |
| --- | --- | --- |
| EGS versus CTRL | Overall | **0.023** |
|  | Stomach | **0.001** |
|  | Ileum | 0.364 |
|  | Caecum | 0.442 |
|  | Colon | 0.115 |
|  | Faeces | 0.146 |
| EGS versus CoG | Faeces | **0.008** |
| CoG versus CTRL | Faeces | **0.006** |

**Table S8; Numbers of differentially abundant (increased, decreased and total) taxa, at phylum, class, order, family, genus and phylotype levels. Comparisons are EGS vs CTRL, EGS vs CoG and CoG vs CTRL groups, overall (data pooled for all sites) and for 5 paired GI site comparisons.**

|  |  | **EGS vs CTRL** | | | | | | **EGS vs CoG** | **CoG vs CTRL** |  |
| --- | --- | --- | --- | --- | --- | --- | --- | --- | --- | --- |
|  |  | **Overall** | **Stomach** | **Ileum** | **Caecum** | **Colon** | **Faeces** | **Faeces** | **Faeces** | |
| Phylum | Increased | 9 | 3 | 4 | 1 | 6 | 1 | 6 | 1 | |
|  | Decreased | 2 | 1 | 1 | 0 | 0 | 1 | 3 | 2 | |
|  | Total | 11 | 4 | 5 | 1 | 6 | 2 | 9 | 3 | |
| Class | Increased | 25 | 4 | 8 | 10 | 15 | 8 | 13 | 5 | |
|  | Decreased | 5 | 4 | 8 | 3 | 1 | 2 | 9 | 2 | |
|  | Total | 30 | 8 | 16 | 13 | 16 | 10 | 22 | 7 | |
| Order | Increased | 56 | 15 | 16 | 38 | 39 | 25 | 45 | 11 | |
|  | Decreased | 24 | 14 | 32 | 3 | 2 | 5 | 9 | 10 | |
|  | Total | 80 | 29 | 48 | 41 | 41 | 30 | 54 | 21 | |
| Family | Increased | 137 | 27 | 23 | 102 | 89 | 78 | 112 | 23 | |
|  | Decreased | 38 | 32 | 55 | 10 | 12 | 14 | 16 | 17 | |
|  | Total | 175 | 59 | 78 | 112 | 101 | 92 | 128 | 40 | |
| Genus | Increased | 310 | 68 | 50 | 208 | 161 | 147 | 212 | 51 | |
|  | Decreased | 91 | 76 | 106 | 29 | 28 | 36 | 42 | 26 | |
|  | Total | 401 | 144 | 156 | 237 | 189 | 183 | 254 | 77 | |
| Phylotypes | Increased | 560 | 122 | 108 | 305 | 243 | 229 | 365 | 85 | |
|  | Decreased | 186 | 139 | 160 | 54 | 43 | 82 | 91 | 82 | |
|  | Total | 746 | 261 | 268 | 359 | 286 | 311 | 456 | 167 | |
